# Supplementary material for: Mutations mark cell lineages and sectors in flowers of a woody angiosperm
Source: PLoS Genet. 2025 Aug 18;21(8):e1011829. doi: 10.1371/journal.pgen.1011829 (PMC12370204; doi:10.1371/journal.pgen.1011829)
Supplement: S1 Fig — (PDF) [file pgen.1011829.s001.pdf]

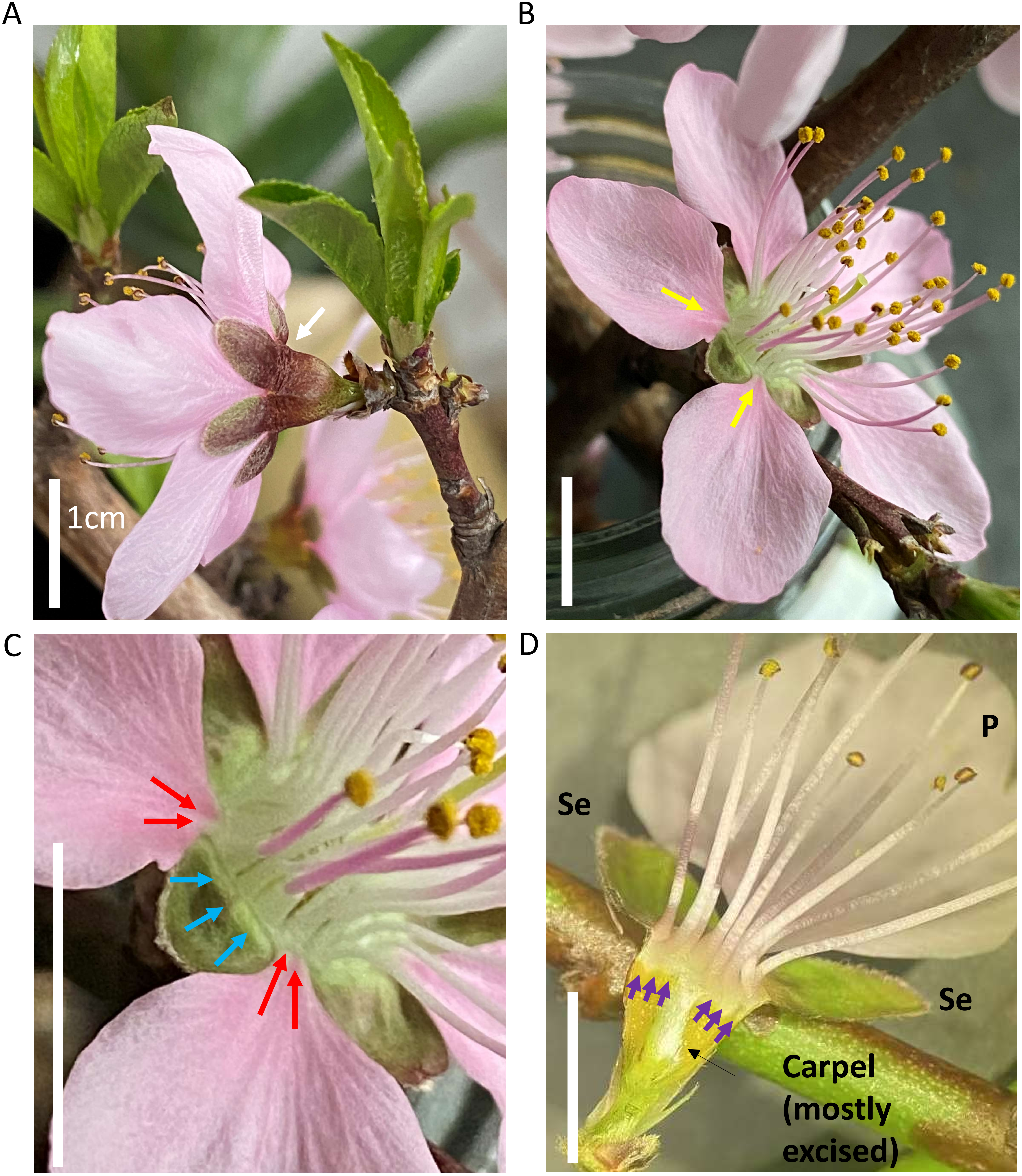

S1_Fig. Peach floral structure showing sepal, petal, stamen, and carpel connections for focal peach tree DHQ1. A) Sepals connect to floral cup continuously along perimeter of radial disc (see white arrow), lack articulation, and are retained at anthesis. B) Petals connect at discrete intervals (yellow arrows) around the perimeter of the floral cup, are articulated, and abscise at the junction following anthesis. C) Stamens emerge from cup at base of each petal (red arrows) as well as from cup below each sepal (blue arrows). D) Sagittal section showing two sepals (Se), the intervening petal (P), and stamens arising from the cup. Here, three stamens (purple arrows) arise at the base of each sepal. Scale bar = 1cm. (All photo credits: B. Traw).
