# Supplementary material for: Mutations mark cell lineages and sectors in flowers of a woody angiosperm
Source: PLoS Genet. 2025 Aug 18;21(8):e1011829. doi: 10.1371/journal.pgen.1011829 (PMC12370204; doi:10.1371/journal.pgen.1011829)
Supplement: S2 Fig — (PDF) [file pgen.1011829.s002.pdf]

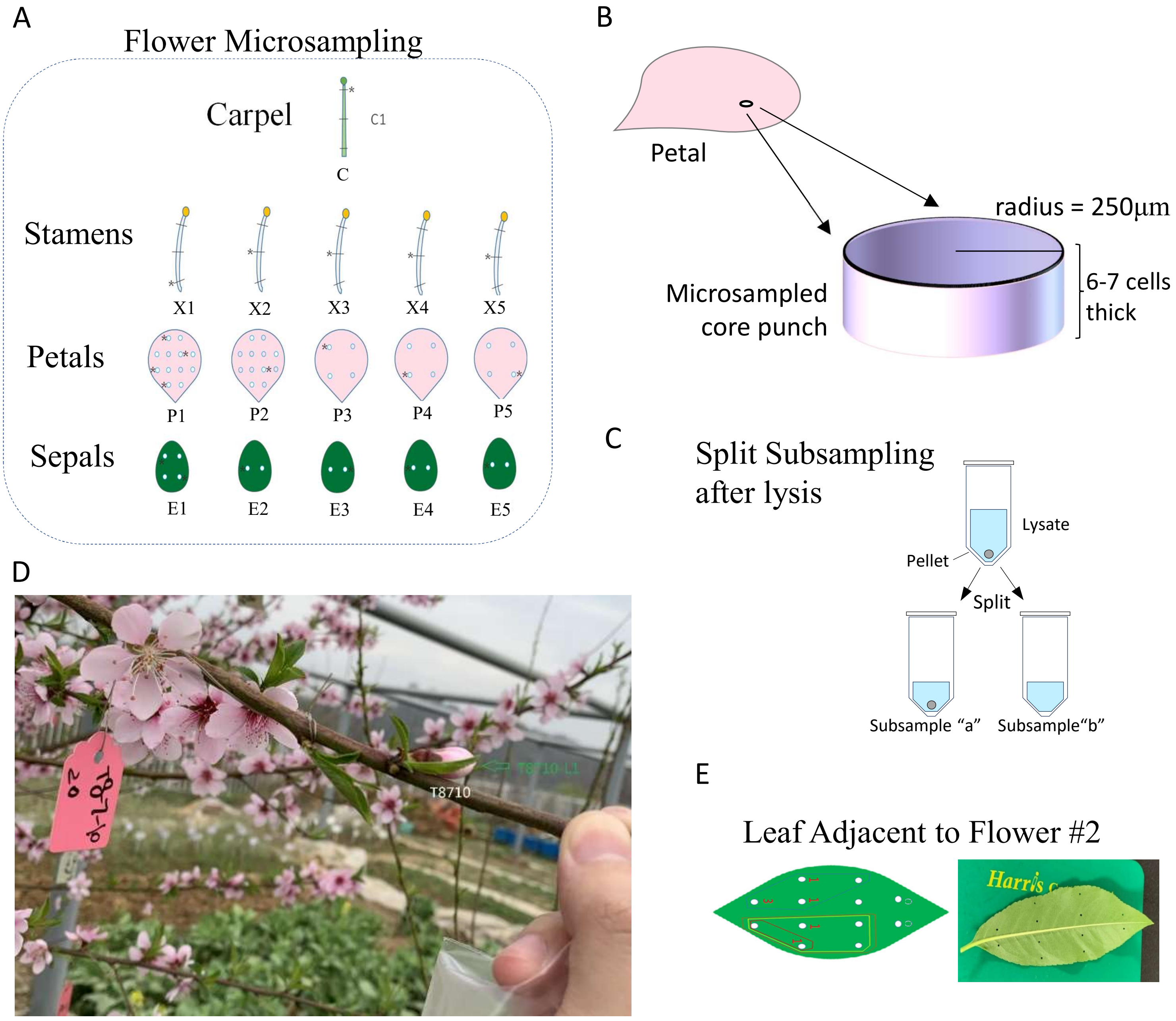

S2_Fig. Peach flower microsampling and split subsampling. **A)** Micropunch disks taken from sepals (E1-5), petals (P1-5). Dissection of five selected stamens (X1-5) and the single carpel (C1) into three samples each by razor blade. **B**) Estimation of cell numbers yielding DNA in punched microsamples of peach petals. Epidermal cell surface area of petal estimated as π*5^2 ≈ 78µm^2 based on radius of 5µm. Given petal thickness of 6-7 cells, total cells per micropunch disk is approximately 6*π*250^2/78 ≈ 1.5*10^4 cells. If lysis only releases cell contents along the perimeter of the disk, then estimate is approximately 1000 sampled cells (6*500*π/10 ≈ 942 cells). **C**) To obtain split subsamples, twenty microsamples (indicated by asterisks) were processed to the lysate stage and then split into two equal fractions and sequenced independently for Flower #2 only to assess the technical repeatability of mutation calls. **D**) Flower #2 on Subbranch B1-1-8-7-10 prior to sampling (Photo credit: Y. Li.). **E**) Nearest subtending leaf (B1-1-8-7-10-L1) as sampled July 2019 with 12 microsamples (Photo credit: Y. Li.), for which ten yielded analyzable data (S1_Table).
