# Supplementary material for: Mutations mark cell lineages and sectors in flowers of a woody angiosperm
Source: PLoS Genet. 2025 Aug 18;21(8):e1011829. doi: 10.1371/journal.pgen.1011829 (PMC12370204; doi:10.1371/journal.pgen.1011829)
Supplement: S3 Fig — (PDF) [file pgen.1011829.s003.pdf]

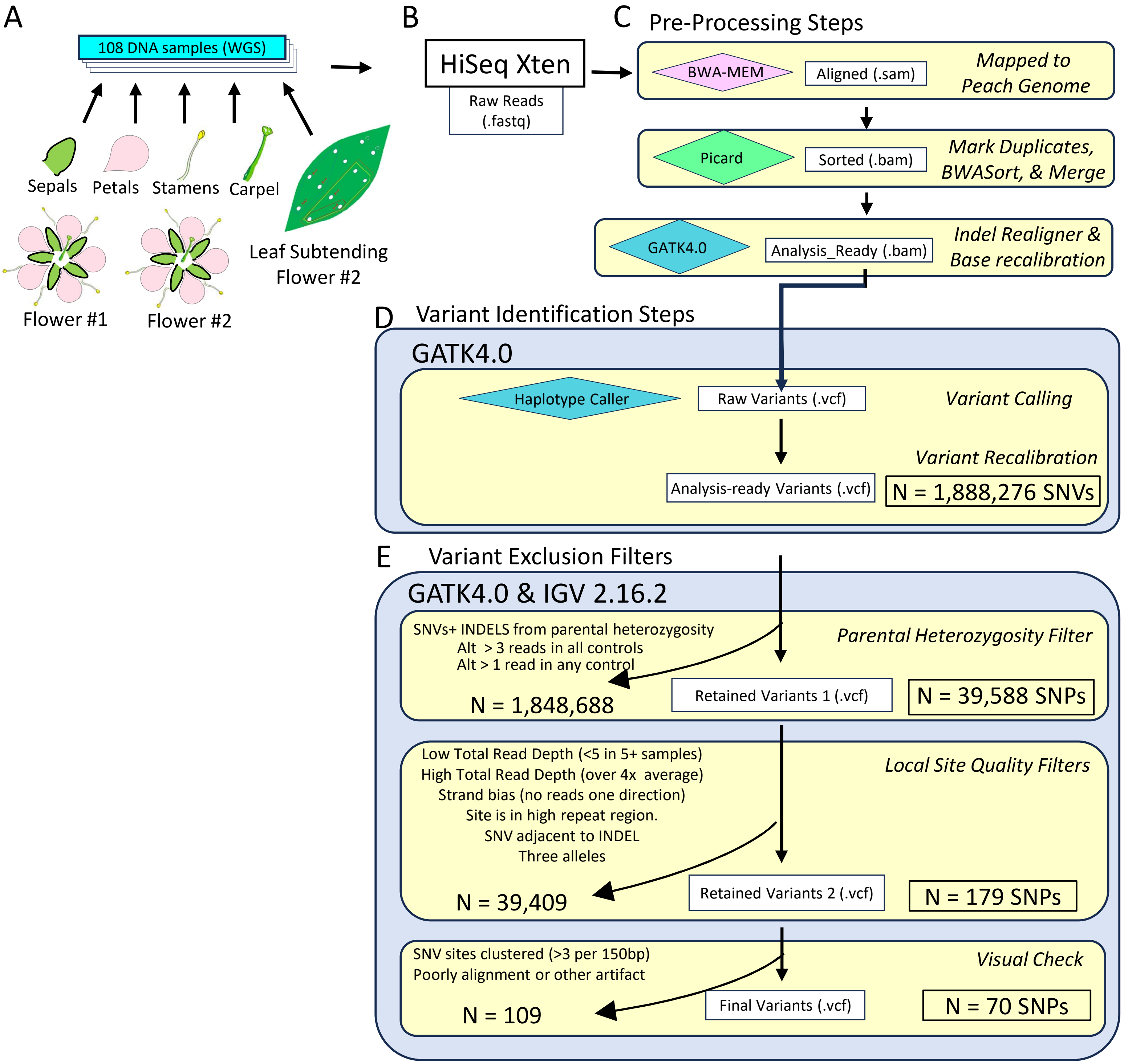

S3_Fig. Flowchart of mutation identification pipeline. A) Collection of 108 independent DNA samples from sepals, petals, stamens, and carpel of two flowers and subtending leaf. B) HiSeq Xten platform generation of raw reads. C) Three pre-processing steps beginning with BWA-MEM to map the reads to the peach genome, followed by Mark Duplicates in Picard to exclude non legitimate reads, and Indel Realigner in the GATK4.0 workbench to correctly mark areas at indel sites relative to the published peach genome. D) All 108 analysis ready BAM files were then processed together to identify 1,888,276 raw SNV variants. E) Three major exclusion filters were then applied sequentially. First, 1,848,688 differences that were due to SNV and INDELS reflecting parental heterozygosity were identified and excluded, leaving 39,588 SNPs. Of these 39,588 SNPs, a large portion (39,409) were in low quality sites (total read depth too low or too high, strand biased, high repeat region, adjacent to an INDEL, a single low genome quality stamen sample, or three alleles at the SNV) and were excluded, leaving 179 SNPs. Finally a visual check was made for the remaining 179 SNPs. Of these 179 SNPs, 109 occurred in SNV site clusters or exhibited poor overall read alignment and were excluded, leaving 70 high quality candidate *de novo* mutations in the flowers and subtending leaf (S2 & S3 Tables).
