# Supplementary material for: Mutations mark cell lineages and sectors in flowers of a woody angiosperm
Source: PLoS Genet. 2025 Aug 18;21(8):e1011829. doi: 10.1371/journal.pgen.1011829 (PMC12370204; doi:10.1371/journal.pgen.1011829)
Supplement: S4 Fig — (PDF) [file pgen.1011829.s004.pdf]

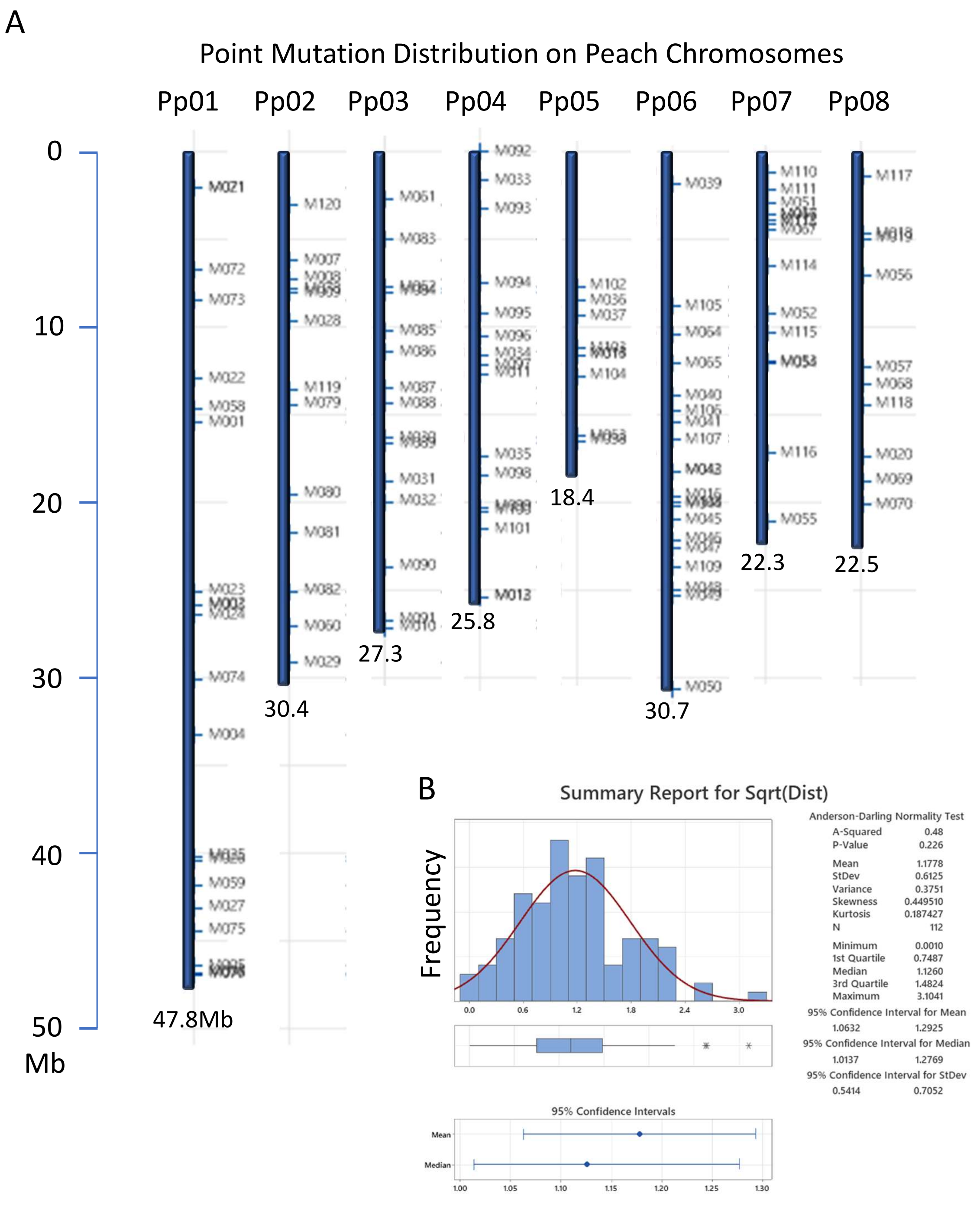

S4_Fig. Distribution of point mutations in the peach genome. Shown are point mutations for Flower #1 (#M1 – M#20), Flower #2 (#M21-#M57), adjacent leaf (#M58-#M70), and older branch leaves (#M71-#M120), as indicated (S2 Table). A) Locations of the mutations on the eight chromosomes. B) Frequency distribution of average distance between adjacent mutations following square root transformation. Average distance between mutations is 1.7MB. Distribution of distances does not differ significantly from normality by Anderson-Darling test (P= 0.226).
