# Supplementary material for: Mutations mark cell lineages and sectors in flowers of a woody angiosperm
Source: PLoS Genet. 2025 Aug 18;21(8):e1011829. doi: 10.1371/journal.pgen.1011829 (PMC12370204; doi:10.1371/journal.pgen.1011829)
Supplement: S5 Fig — (PDF) [file pgen.1011829.s005.pdf]

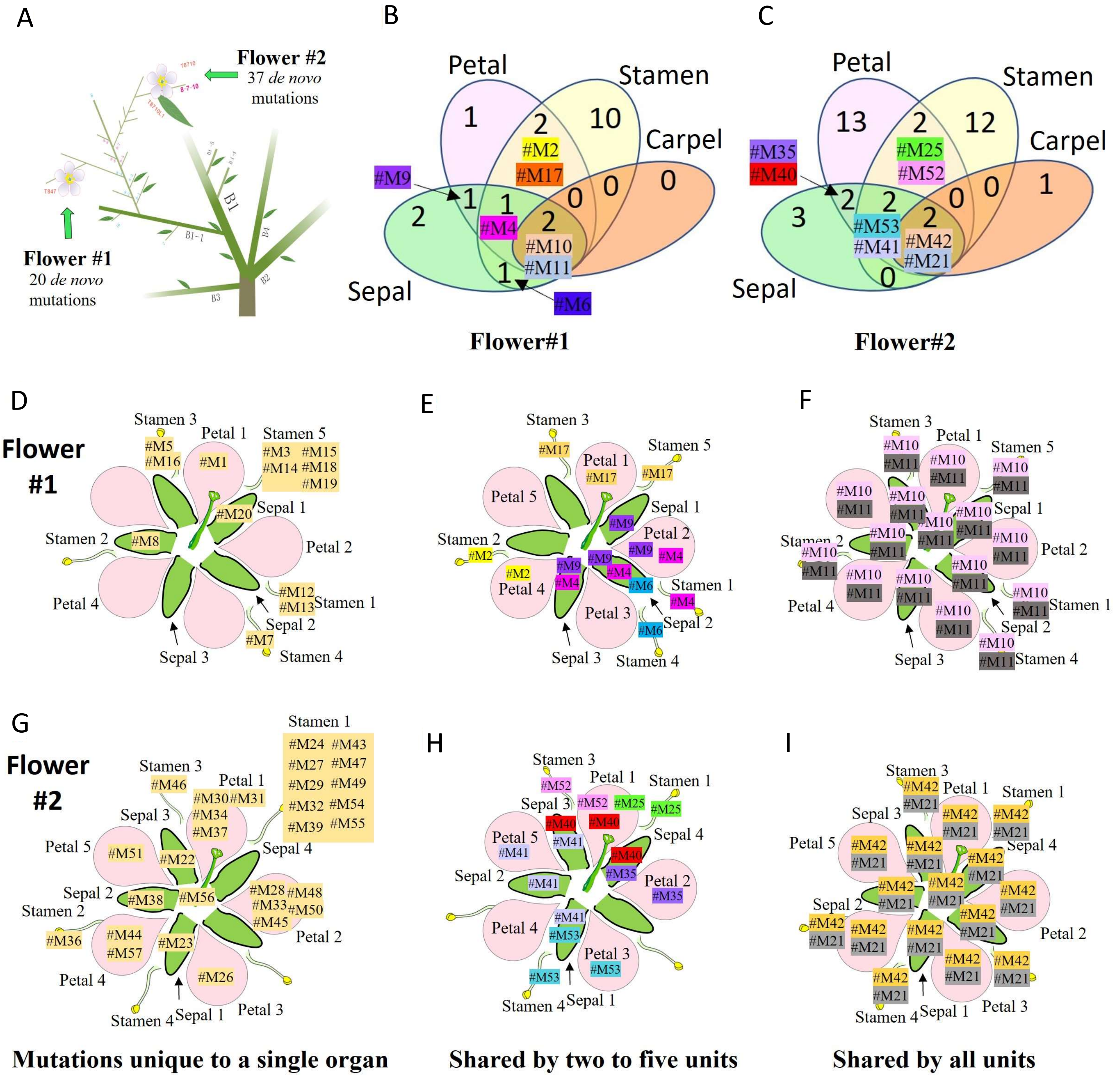


**S5_Fig**. Distribution of the 57 *de novo* floral mutations (#M01 - #M57) among flower parts. **A**) Locations of Flower #1 and Flower #2 on Sub-Branch B1-1-8 of Branch B1-1 of Main Branch B1. Venn diagrams showing the relative incidence of mutations in sepal, petal, stamen, and carpel in **B)** Flower #1 and **C)** Flower #2. **D – I)** Floral diagrams showing where mutations were found for **D - F)** Flower #1 and **G – I)** Flower #2, separated by **D, G)** Mutations unique to a single unit of a single organ, **E, H**) Mutations shared by two to five units, and **F, I**) Mutations shared by all units of all organs. Mutation chromosome positions (S2 Table) and base calls in all samples are provided (S3 Table).
