# Supplementary material for: Mutations mark cell lineages and sectors in flowers of a woody angiosperm
Source: PLoS Genet. 2025 Aug 18;21(8):e1011829. doi: 10.1371/journal.pgen.1011829 (PMC12370204; doi:10.1371/journal.pgen.1011829)
Supplement: S6 Fig — (PDF) [file pgen.1011829.s006.pdf]

**A**

| Class | Interpretation | #Tests | % | |
|---|---|---|---|---|
| **True Negative** | Biologically correct | 669 | 90.4 | 97.1% |
| **True Positive** | Biologically correct | 50 | 6.7 | |
| **False Negative** | Lack of sensitivity in detection | 11 | 1.5 | 2.9% |
| **False Positive** | Environmental DNA contamination | 10 | 1.4 | |
| **Total** | | 740 | 100.0 | |

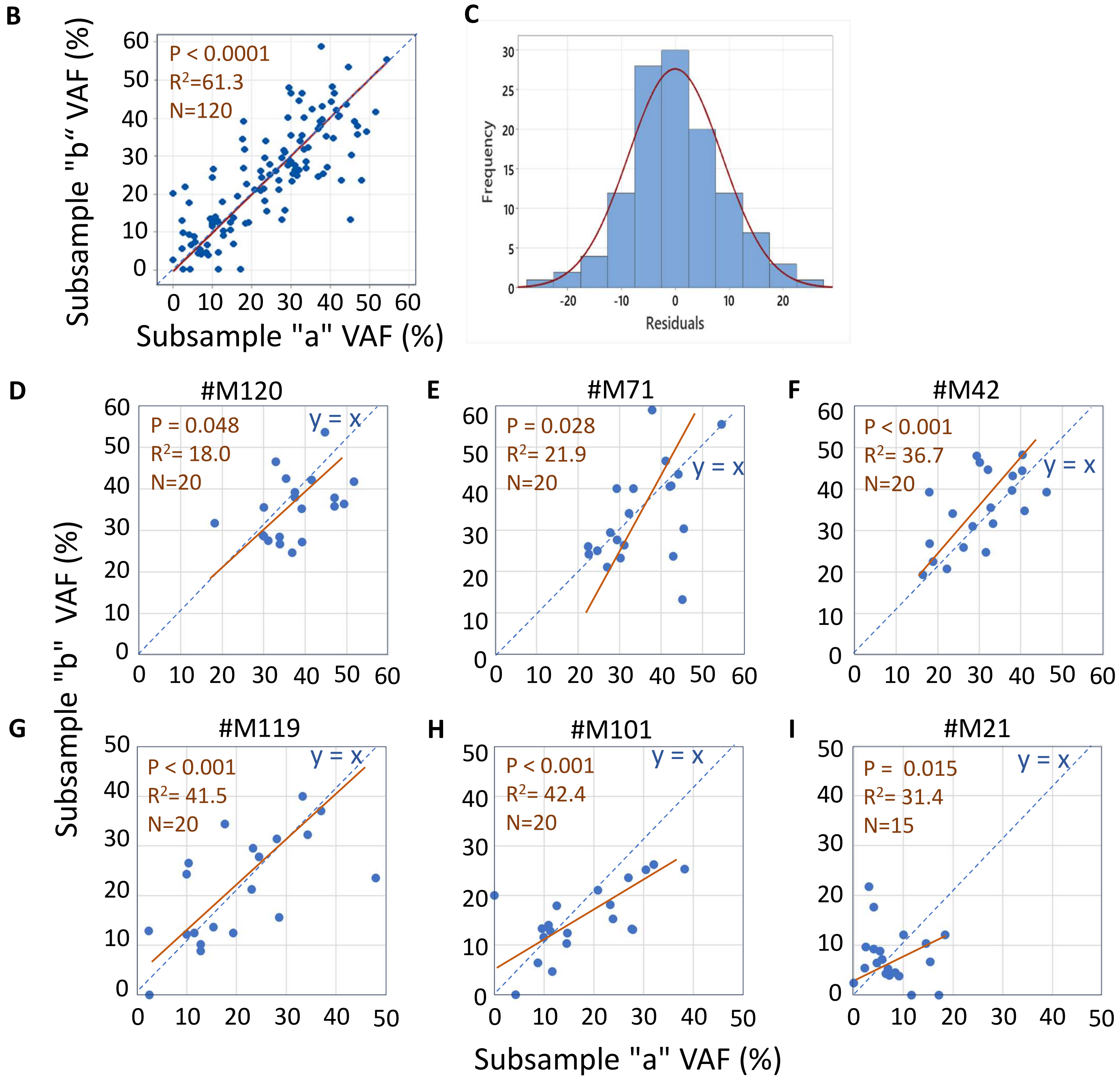


**S6_Fig.** Analysis of paired split subsamples. **A)** Summary of 37 Flower #2 mutations assessed in all 20 pairs (740 total comparisons). Consistency between subsamples in a pair was observed 97.1% of comparisons (S5 Table). **B)** Scatterplot of variant allele frequency between "a" vs "b" subsamples from 20 biological samples of Flower #2 for VAF (%) for the six widespread mutations. **C)** HIstogram showing normal distribution of residuals. **D-I)** Scatterplots shown separately for all six widespread mutations, **D**) #M120, **E**) #M71, **F**)#M42, **G**) #M119, **H**) #M101, and **I**) #M21. Three high and two low outliers in #M21 are included in the scatterplot but excluded from the orthogonal regression (S6 Table). Orthogonal regression (red line), y = x (blue dashed line), Pvalue, regression fit, and sample sizes shown. All raw data provided (S5 & S6 Tables).
