# Supplementary material for: Mutations mark cell lineages and sectors in flowers of a woody angiosperm
Source: PLoS Genet. 2025 Aug 18;21(8):e1011829. doi: 10.1371/journal.pgen.1011829 (PMC12370204; doi:10.1371/journal.pgen.1011829)
Supplement: S7 Fig — (PDF) [file pgen.1011829.s007.pdf]

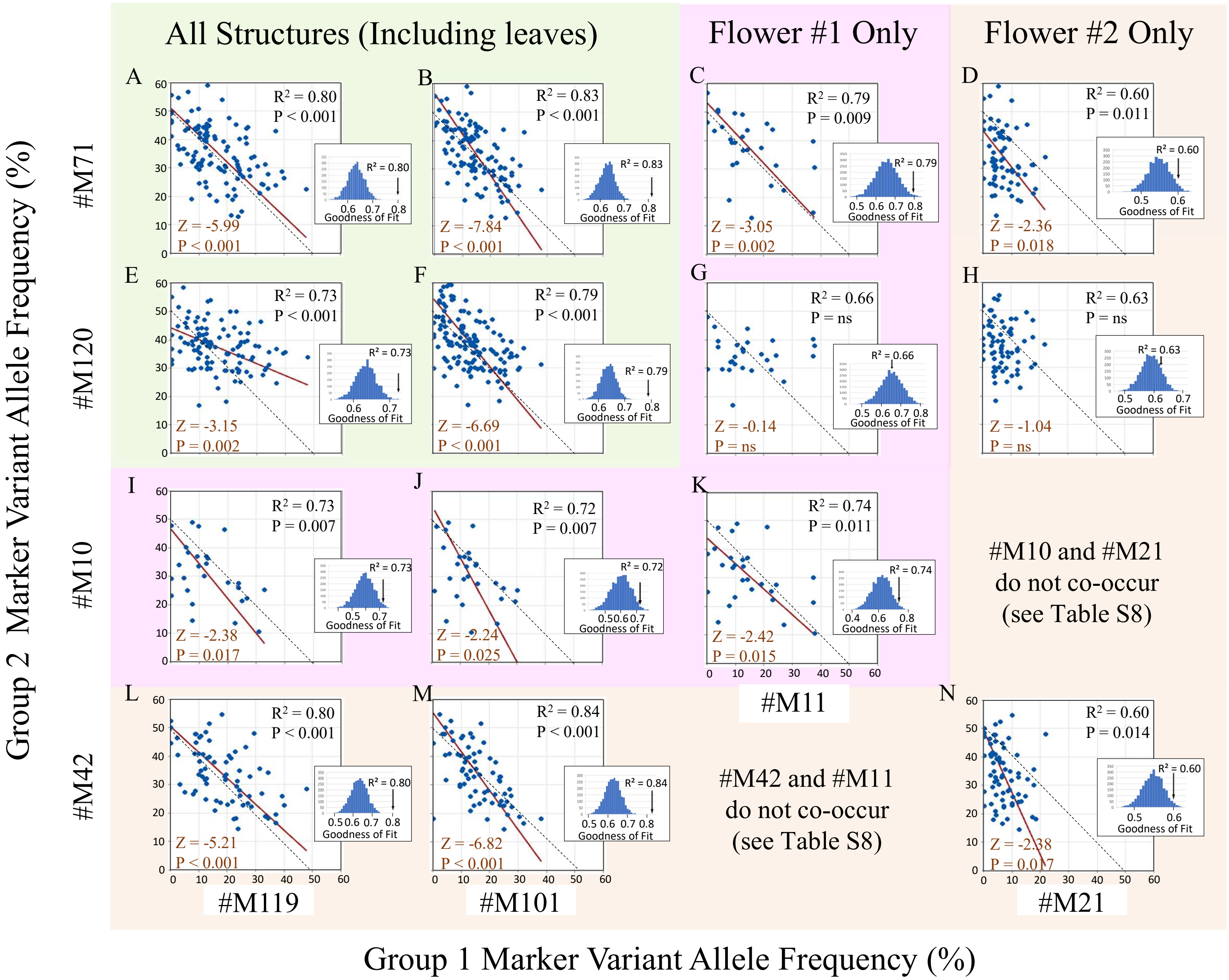

S7_Fig. Negative VAF (%) correlations for Group 1 and Group 2 marker pairs. All possible combinations of the four major Group 1 and four Group 2 marker mutations shown. Dotted lines represent y = 100 - x. All axis are 0 to 60%. Orthogonal regression line with Z value and P value shown (in brown). Inset histograms show observed fit (R^2) to %Group 1 + %Group 2 = 100% line relative to the distribution of possible goodness of fit values obtained from permutation tests (N = 3000) with y and x values paired randomly. VAF (%) of Group 2 Mutation #M71 relative to Group 1 Mutations **A**) #M119, **B**) #M101, **C**) #M11, and **D**) #M21, respectively. VAF (%) of Group 2 Mutation #120 relative to Group 1 Mutations **E**) #M119, **F**) #M101, **G**) #M11, and **H**) #M21, respectively. VAF (%) of Group 2 Mutation #10 of Flower #1 relative to Group 1 Mutations **I**) #M119, **J**) #M101, and **K**) #M11, respectively. VAF (%) of Group 2 Mutation #42 of Flower #2 relative to Group 1 Mutations **L**) #M119, **M**) #M101, and and **N**) #M21, respectively. Two points in subpanel E not shown, (72.3,5.1) and (70.0,4.9) to keep uniform axis length. However, these two values are included in the statistical analyses. Raw data for all plots provided (S7 Table).
