# Supplementary material for: Mutations mark cell lineages and sectors in flowers of a woody angiosperm
Source: PLoS Genet. 2025 Aug 18;21(8):e1011829. doi: 10.1371/journal.pgen.1011829 (PMC12370204; doi:10.1371/journal.pgen.1011829)
Supplement: S8 Fig — (PDF) [file pgen.1011829.s008.pdf]

Positive VAF Correlations Among Mutations Marking the Group 1 Cell Lineage

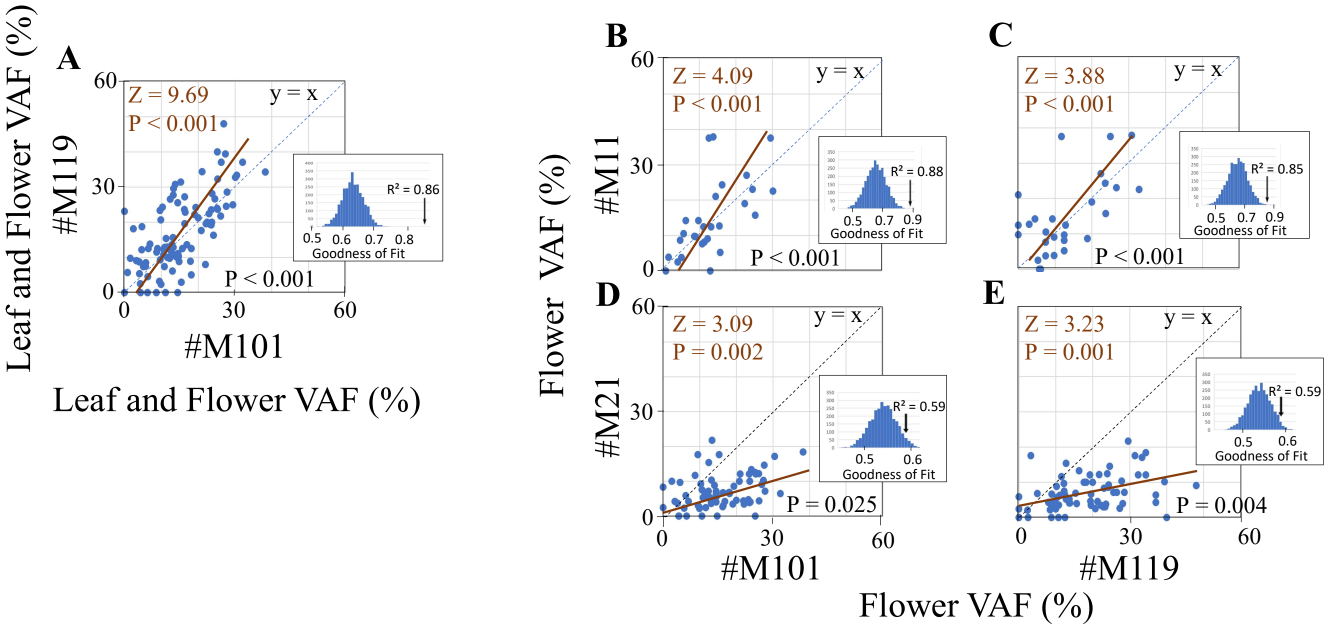

Positive VAF Correlations Among Mutations Marking the Group 2 Cell Lineage

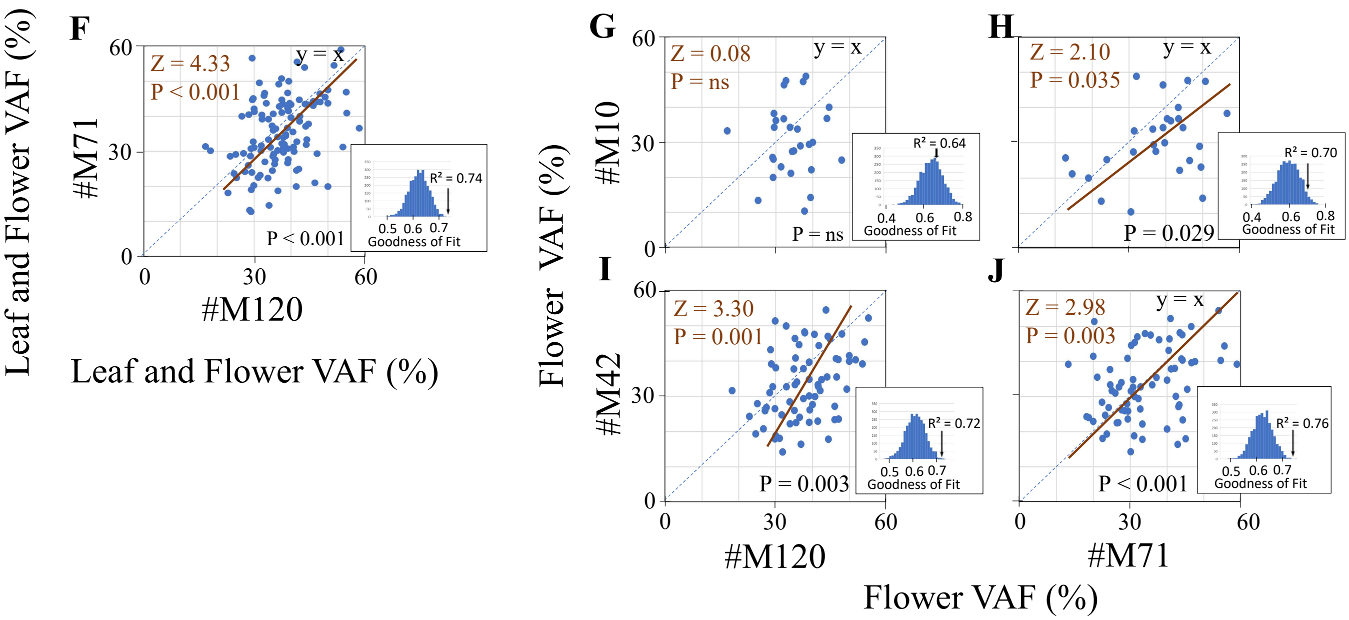

S8_Fig. Positive VAF (%) correlations for Group 1 and Group 2 marker pairs. **A-E**) Mutations #M119, #M101, #M11, and #M21 marking the Group 1 lineage and **F-J**) Mutations #M71, #M120, #M10, and #M42 marking the Group 2 lineage. Dotted lines represent y = x. All axis are 0 to 60%. Orthogonal regression line with Z value and P value are shown (in brown). Inset histograms show observed fit (R^2) of the data to y = x line relative to the distribution of possible goodness of fit values obtained from permutation tests (N = 3000) with y and x values paired randomly. Scatterplots for Group 1 linked mutations including **A**) Subbranch B1-1 Mutation #M119 and #M101. **B, C**) Floral Mutation #M11 of Flower #1 with #M101 and #M119, respectively. **D, E**) Floral Mutation #21 of Flower #2 with #M101 and #M119, respectively.) Scatterplots for Group 1 linked mutations including **F**) Main trunk Mutation #M120 and Subbranch B1-1 Mutation #M71, **G, H**) Floral Mutation #M10 of Flower #1 with #M120 and #M71, respectively. **I, J**) Floral Mutation #M42 of Flower #2 with #M120 and #M71, respectively. Raw data provided (S7 Table).
