# Supplementary material for: Mutations mark cell lineages and sectors in flowers of a woody angiosperm
Source: PLoS Genet. 2025 Aug 18;21(8):e1011829. doi: 10.1371/journal.pgen.1011829 (PMC12370204; doi:10.1371/journal.pgen.1011829)
Supplement: S9 Fig — (PDF) [file pgen.1011829.s009.pdf]

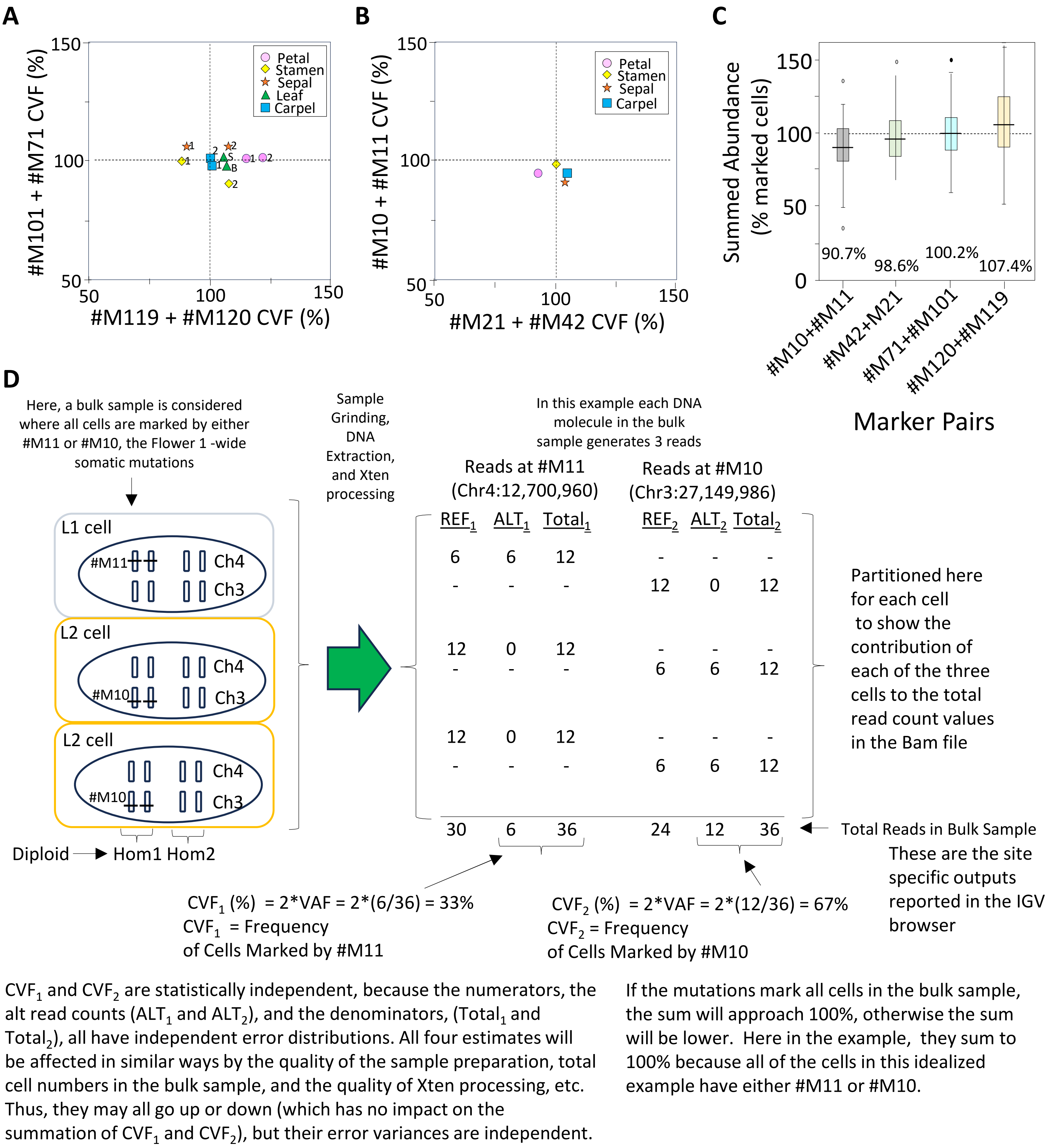

S9_Fig. Estimation of cell variant frequency (CVF) from variant allele frequency (VAF). Pairs of L1 and L2 marker mutations sum to approximately 100% for both A) deep branch mutation L1 and L2 pairs, #M119 plus #M120, and #M101 plus #M71, respectively, and B) flower-wide mutation L1 and L2 pairs, #M21 plus #M42, and #M10 plus #M11, respectively (S8 Table). C) box and whisker plots showing distributions of summed abundances for all samples. #M21 is doubled in both scatterplots to account for its likely origin one cell division after the other mutations D) Relationship between cell numbers, Xten read counts, and cell variant frequency calculations with example of bulk sample analyzed for Flower 1 flowerwide mutations #M10 and #M11, which are on Chromosomes 3 and 4, respectively.
