# Supplementary material for: Mutations mark cell lineages and sectors in flowers of a woody angiosperm
Source: PLoS Genet. 2025 Aug 18;21(8):e1011829. doi: 10.1371/journal.pgen.1011829 (PMC12370204; doi:10.1371/journal.pgen.1011829)
Supplement: S10 Fig — (PDF) [file pgen.1011829.s010.pdf]

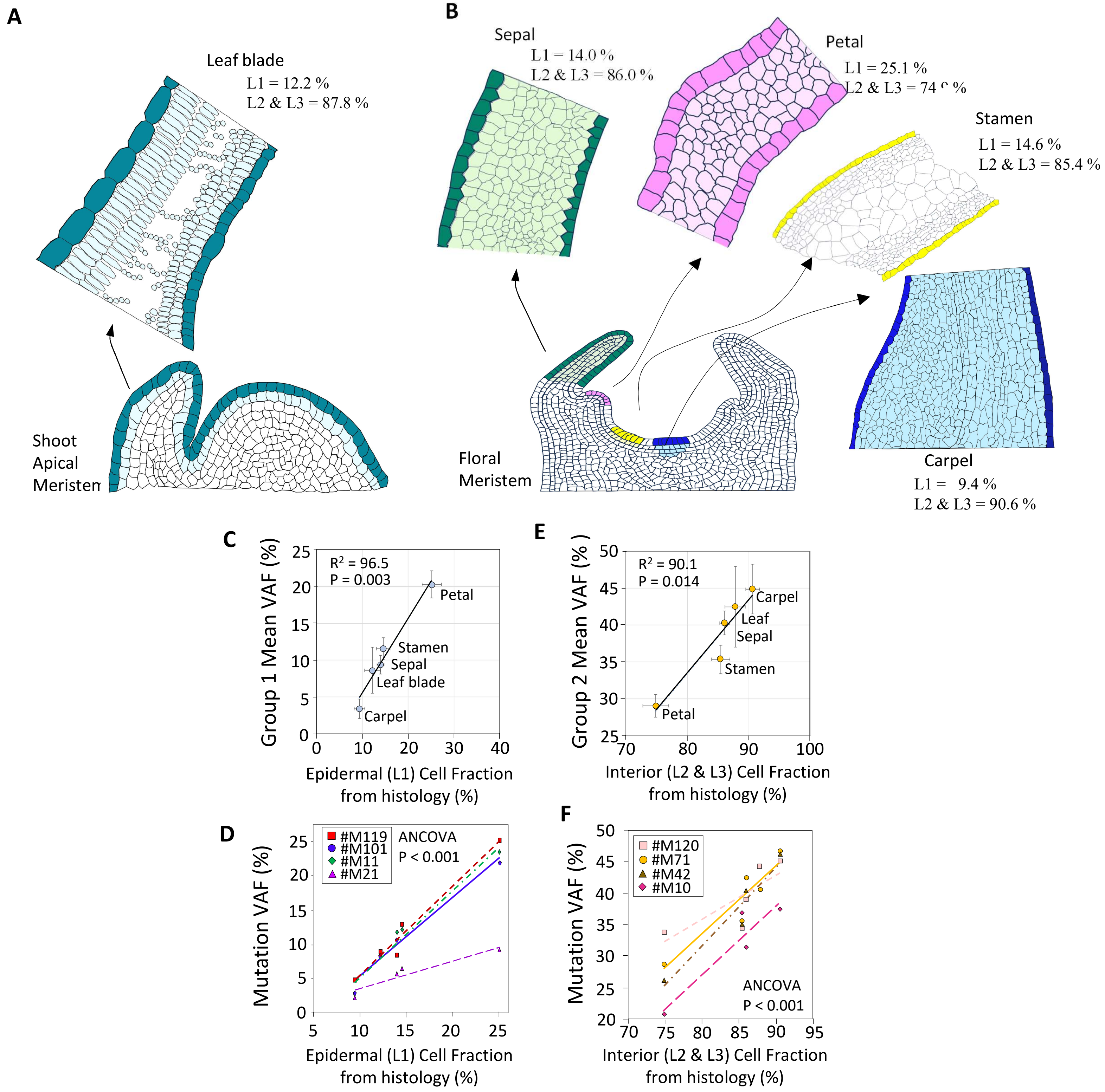

S10_Fig. VAF estimates correspond to epidermal and interior cell populations. Estimated proportion of epidermal cells (L1) to interior cells (L2 and L3 combined) in **A**) leaves and **B**) sepal, petal, stamen,and carpel determined from analysis of published histological cross-sections (S10 & S11 Figs; S9 Table). L2 and L3 are combined to focus on epidermis (L1), and prior evidence that L3 lacks genetic fidelity in peach (Dermen & Stewart 1973). Positive correlation between Group 1 mutation VAF and L1 cell abundance for **C**) the combined Group 1 VAF estimate and **D**) individual VAF estimates for #M119 and #M101, #M11, #M21, analyzed by ANCOVA (P value from L1 main effect shown). Positive correlation between Group 2 mutation VAF and Interior (L2 + L3) cell abundance for **E**) the combined Group 2 VAF estimate and **F**) individual VAF estimates for #M119 and #M101, #M11, #M21 analyzed by ANCOVA. Error bars in C and E represent range of raw data (x-axis) and 95% confidence interval (y-axis).
