# Supplementary material for: Mutations mark cell lineages and sectors in flowers of a woody angiosperm
Source: PLoS Genet. 2025 Aug 18;21(8):e1011829. doi: 10.1371/journal.pgen.1011829 (PMC12370204; doi:10.1371/journal.pgen.1011829)
Supplement: S11 Fig — (PDF) [file pgen.1011829.s011.pdf]

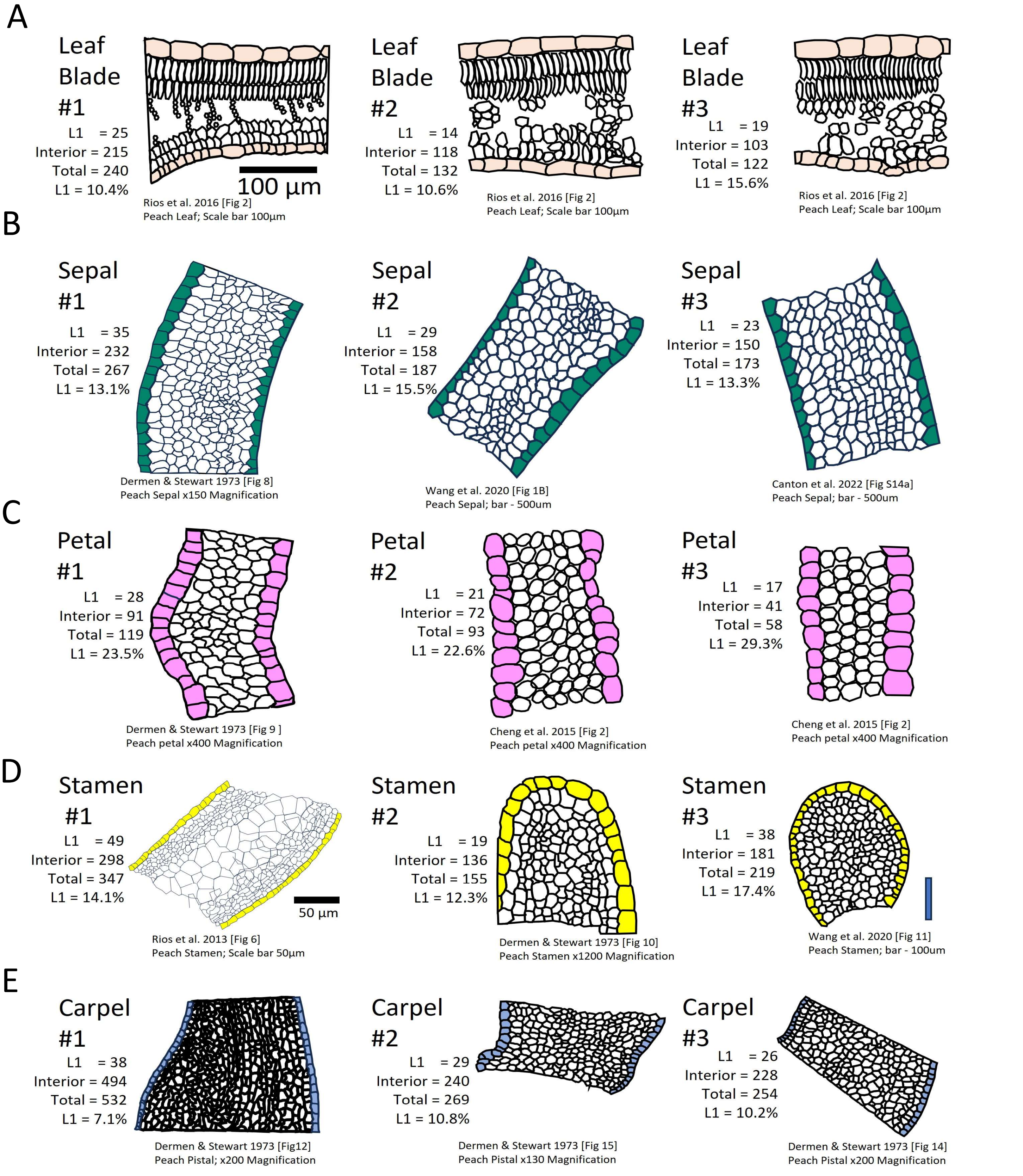

S11_Fig. Epidermal and interior cell counts in *Prunus persica* A) leaf blade, B) sepal, C) petal, D) stamen, and E) carpel. Image sources for A) leaf (Fig 2 in Rios et al 2016), B) sepal (Dermen & Stewart 1973, Wang et al. 2020, Canton et al. 2022), C) petal (Dermen & Stewart 1973, Cheng et al. 2015), D) stamen (Rios et al. 2013, Dermen & Stewart 1973, Wang et al. 2020), and E) carpel (Dermen & Stewart 1973), as indicated below each drawing. All images longitudinal sections, with the exception of leaf (a transverse section near midrib). Outlined in graphic tools (Powerpoint 2023). Cell counts in ImageJ (settings: 8-bit, circularity > 0.3) shown for interior and epidermal cells . M, Mesophyll; pmc, pollen mother cell. Data summary provided (S8 Table).
