# Supplementary material for: Mutations mark cell lineages and sectors in flowers of a woody angiosperm
Source: PLoS Genet. 2025 Aug 18;21(8):e1011829. doi: 10.1371/journal.pgen.1011829 (PMC12370204; doi:10.1371/journal.pgen.1011829)
Supplement: S12 Fig — (PDF) [file pgen.1011829.s012.pdf]

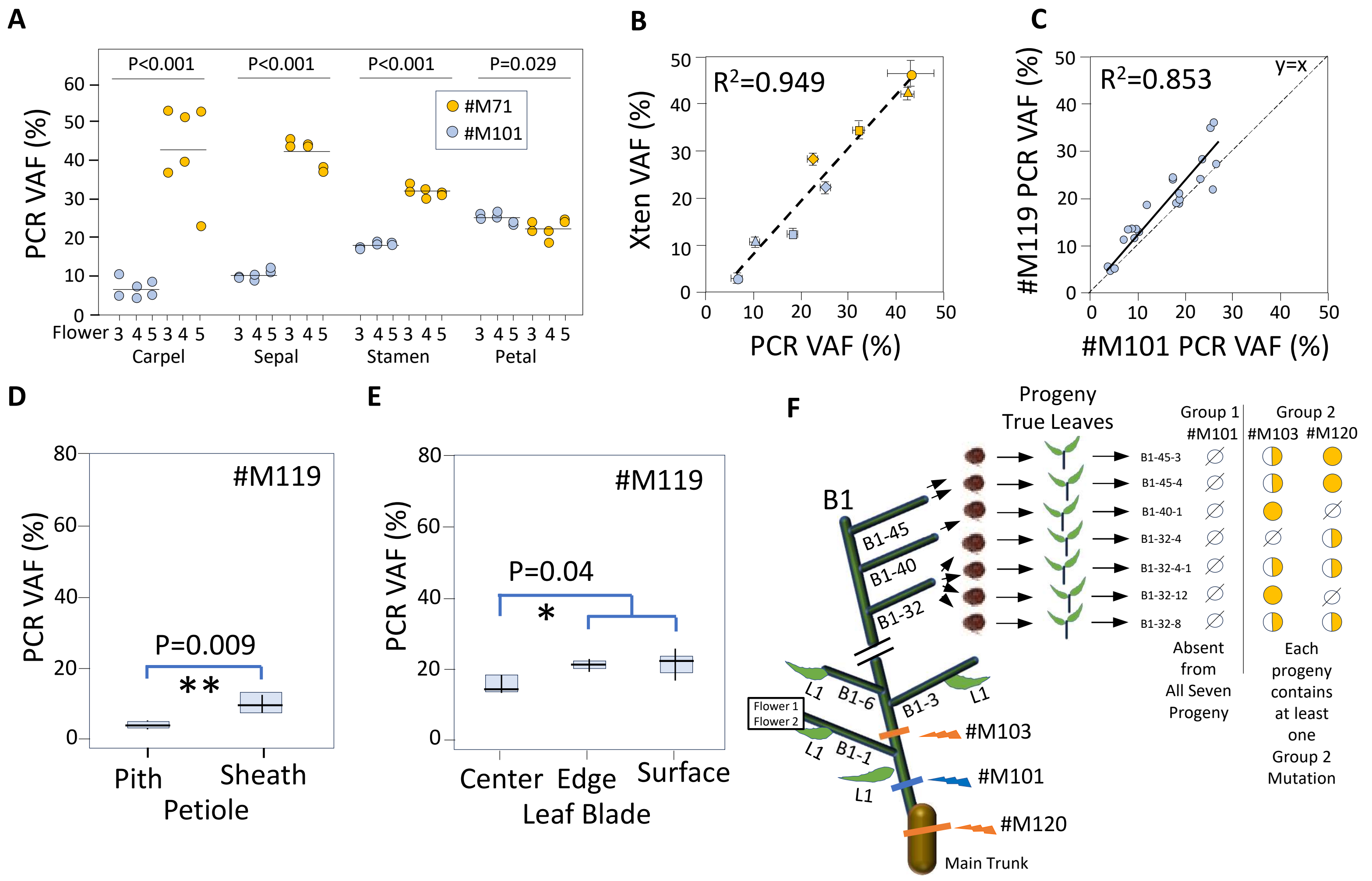

S12_Fig. Group 1 and Group 2 mutations in additional flowers, leaves, and progeny. **A**) PCR-based quantification of #M71 and #M101 variant allele frequencies (%) in three flowers (Flowers# 3-5). P values from one-way ANOVA. **B**) High correlation in Xten and PCR estimates of marked cell frequencies (%), showing #M101 (blue) and #M71 (orange) in carpel (circles), sepals (triangles) stamens (squares), and petals (diamonds). **C**) High correlation between #M119 and #M101 PCR estimation in flower samples from Flowers 3,4, and 5. Variant allele frequency (%) of #M119 mutation in **D)** petiole pith versus sheath and **E**) leaf blade surface scrape versus edge versus center. **F**) Seven progeny sampled for Group 1 and Group 2 markers. All seven possess a Group 2 mutation (#M103 or #M120) indicating the presence of the mutation marked Group 2 lineage cells, while none have the #M101 mutation and therefore lack a genetic contribution of the Group 1 cell lineage. Raw data provided (S11 Table). In some cases, a deviation in read counts from 50:50 was observed and is expected due to the errors associated with read estimation, as verified with our split subsamples (S6 Table).
