# Supplementary material for: Mutations mark cell lineages and sectors in flowers of a woody angiosperm
Source: PLoS Genet. 2025 Aug 18;21(8):e1011829. doi: 10.1371/journal.pgen.1011829 (PMC12370204; doi:10.1371/journal.pgen.1011829)
Supplement: S13 Fig — (PDF) [file pgen.1011829.s013.pdf]

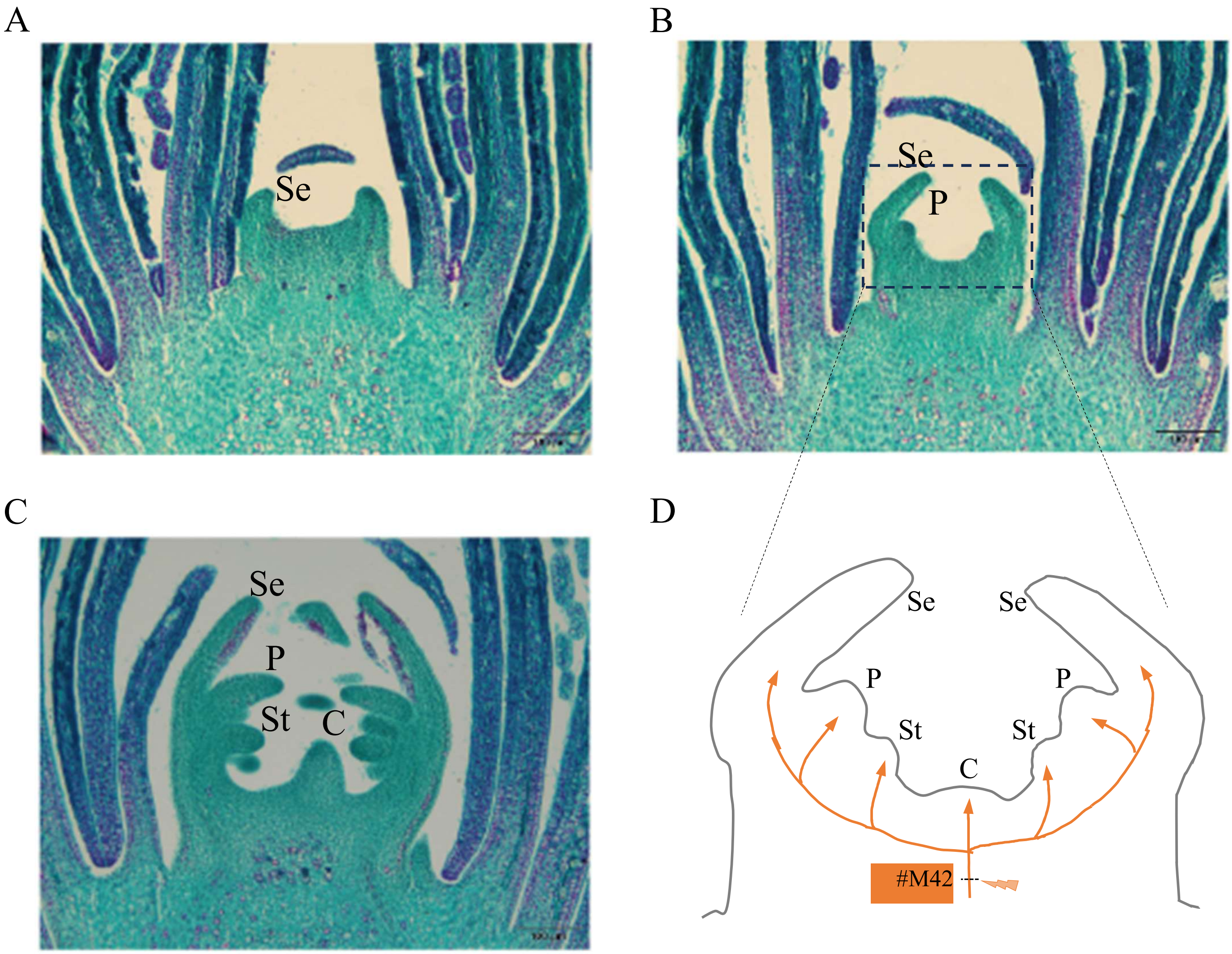

S13_Fig. Floral bud cross-section chronology. **A-C**) Stages of development in peach flower buds. Cross-sections of *Prunus* flower buds on **A**) 16 June, **B**) 22 June, **C**) 31 June, showing primordial development of Sepals (Se), Petals (P), Stamens (St), and Carpel (C). Images A-C from a published work (Xu et al., 2022) and reprinted here under the terms of the Creative Commons Attribution License (CC BY). **D**) Floral meristem cross-section diagram showing example of Mutation #M42 which is present in all 70 samples collected from the five sepals, five petals, five stamens, and carpel of Flower #2 (S7 Table).
