# Supplementary material for: Mutations mark cell lineages and sectors in flowers of a woody angiosperm
Source: PLoS Genet. 2025 Aug 18;21(8):e1011829. doi: 10.1371/journal.pgen.1011829 (PMC12370204; doi:10.1371/journal.pgen.1011829)
Supplement: S14 Fig — (PDF) [file pgen.1011829.s014.pdf]

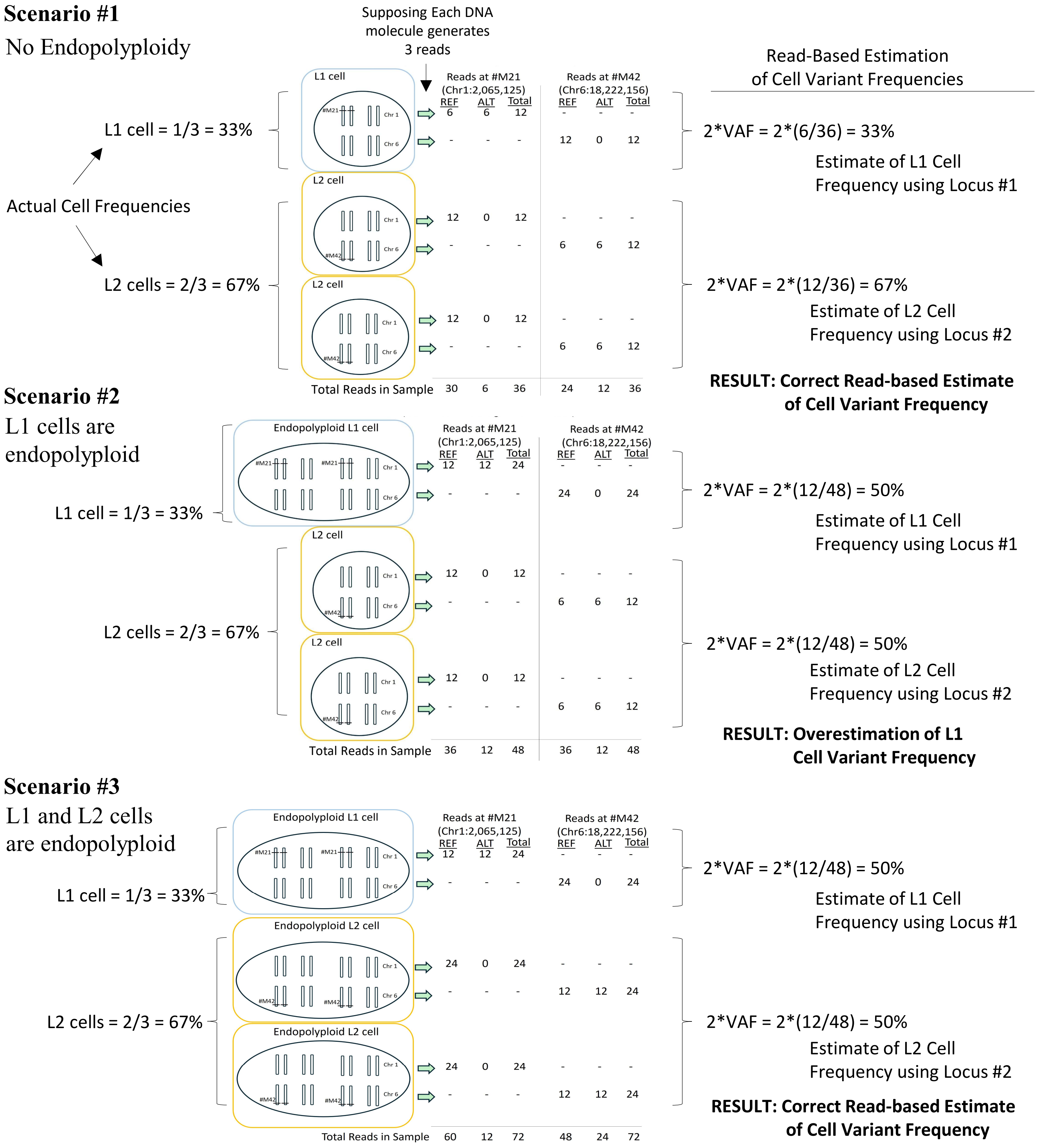

S14_Fig. Effect of endopolyploidy on read-based estimates of cell variant frequency. Consideration of no endopolyploidy (Scenario #1), endopolyploidy of L1 only (Scenario #2) or endopolyploidy in both L1 and L2 (Scenario #3). Shown are three hypothetical cells including one from L1 (epidermis) and two from L2 (interior), where each DNA molecule generates three reads in the relationship of expected read counts to cell frequencies for marker mutations #M21 (Group 1) and #M42 (Group 2).
