# Supplementary material for: Mutations mark cell lineages and sectors in flowers of a woody angiosperm
Source: PLoS Genet. 2025 Aug 18;21(8):e1011829. doi: 10.1371/journal.pgen.1011829 (PMC12370204; doi:10.1371/journal.pgen.1011829)
Supplement: Attachment — Submitted filename: Response to Reviewers_last.docx [file pgen.1011829.s029.docx]

Reviewer #1: This study investigates floral development in peach by tracking genome-wide de novo mutations in two individual flowers. Specifically, the authors ask whether shared mutations define radial sectors that span sepals or sepals and adjacent petals, whether carpels share mutations with radial sectors, and whether mutation patterns can be used to estimate the number of founder cells in the floral anlagen. The results are intriguing: the authors found that certain mutations are shared between stamens and sepals, but not petals, and that each flower harbored at least two mutations spanning the entire floral structure. These findings are potentially significant. However, the methodology, data presentation, and interpretation require clarification. A major concern lies in the interpretation of SNVs observed at the cell population level, particularly in the context of cell lineage. Since the study does not clearly state what type of stem cell dynamics or lineage behavior is assumed, it is difficult to assess the implications of the observed mutation patterns. The authors should either clarify their assumptions regarding cell lineage and developmental dynamics, or incorporate a mathematical model to bridge the gap between population-level mutation data and cellular-level developmental processes.

We thank the reviewer for suggesting improvements in these areas. To clarify the interpretation of cell lineage and developmental dynamics, we have added paragraphs to the Introduction (Lines 75-94) and Discussion (Lines 292-303). We now cite the excellent new literature (Tomimoto and Satake, 2023; Tomimoto and Satake, 2024; Satake et al., 2024) and convey more clearly the classic work in peach cytochimeric lines (Dermen 1953 and Dermen and Stewart 1973). We thank the Reviewer for helping us convey these lineage expectations in peach.

Specifically, we added these two paragraphs to the Introduction (Lines 75-94):

“Assessment of constitutional mosaicism is possible in this model plant because both branch and floral meristems of peach retain highly conserved cellular organization, where L1 cell lineages, those forming the epidermal layer, divide only in the anticlinal direction, whereas L2 cells divide both anticlinally and periclinally (Dermen, 1953; Dermen and Stewart, 1973). The classic histology studies of peach cytochimeras established both the complete genetic isolation of L1 and L2 and the co-propagation of these two major lineages into all peach axial meristems. This previous work also established with rigor that the L1 layer has only one cell thickness in aerial peach parts and that the proportion of L1 derived (epidermal) cells in leaves and floral parts is low, ranging from 1:10 (leaves, sepals, carpels) to 1:4 (petals) relative to L2, from all of the internal leaf and floral structures are derived (Dermen, 1953; Dermen and Stewart, 1973; Cheng et al., 2015). Thus, the expected VAF (%) of a somatic mutation in L1 is not predicted to exceed 10% in leaves, sepals, or carpels or 25% in petals, under the simplest assumption of uniform ploidy in the L1 and L2 cell populations.

Whether there is a genetic lottery among sublineages within L1 or L2 in terms of who populates the new axiallary meristems is an interesting unanswered question. If there are multiple L1 cell sublineages present in each meristem, then the VAF (%) values for a particular L1 somatic mutation could either be fractionally lower in distal tissues (if each L1 sublineage is propagated into each axial meristem, i.e. the sublineages are conserved) or the max L1 value or nothing (if only one L1 sublineage is propagated into each axial meristem, i.e. the sublineage diversity is replaced by the single successful sublineage). Recent studies have indicated evidence for both models of cell propagation depending upon the tree species considered (Tomimoto and Satake, 2023; Tomimoto and Satake, 2024; Satake et al., 2024).”

Furthermore, in the Discussion we have added a the following paragraph (Lines 292-303):

“Recent studies have indicated evidence for both two different models of cell propagation (conserved versus replaced) depending upon the tree species considered (Tomimoto and Satake, 2023; Tomimoto and Satake, 2024; Satake et al., 2024). If there are multiple L1 cell sublineages present in each meristem, then the VAF (%) values for a particular L1 somatic mutation could either be fractionally lower in distal tissues (if each L1 sublineage is propagated into each axial meristem, i.e. the sublineages are conserved) or the max L1 value or nothing (if only one L1 sublineage is propagated into each axial meristem, i.e. the sublineage diversity is replaced by the single successful sublineage). Here in peach (Fig. 4E), the pattern appears more consistent with replacement where first the unmarked L1 was replaced by the #M101 marked L1, and then subsequently that was in turn was replaced by the #M101+#M119 marked L1. While these two mutations do not support a genetic lottery among sublineages of L1 across the branches studied, more sampling is needed to establish which pattern is more common in peach and other angiosperms.”

The following are more specific comments:

Lines 103–108:
It seems that the time elapsed since the bifurcation of flowers 1 and 2 from the stem cell population is relatively short—likely several weeks or months.

This is correct. The developmental context of the two flowers was roughly equivalent. We now state in the Methods (Lines 416-421) that “Sub-branch 1-1-8 appeared in 2017 and then the two sub-branches (1-1-8-4-7 and 1-1-8-7-10) both emerged in June - July, 2018. The two flowers (1-1-8-4-7-T) and 1-1-8-7-10-T), referred to as Flowers #1 and #2, were collected in May, 2019. Counting from the main trunk, these flowers shared four meristem initiations in common and were at the 21^st^ and 27^th^ nodes, respectively.”

Given that 70 de novo point mutations were detected, what would this imply for the de novo mutation rate per year? Please provide an estimate of the mutation rate based on your data and compare it with previously reported rates in plants or other systems.

We thank the reviewer for the suggestions and have added a new table that compares our observed mutation numbers relative to other studies (Table S13). We have revised the Discussion (Lines 354-361) to address this issue more clearly. We say the following:

“With respect to other studies, our mutation numbers per biological sample reported here for flowers (~0.7) are at the low end of the range observed in other studies of woody angiosperm tissues, where the average is around 4 mutations per sample, with substantial variation among studies (Table S13). Notably, none of the prior studies have explored differences among floral parts. Our stringent filtering of candidates in the mutation identification pipeline may also contribute to the low estimate. Future studies should not filter candidates by requiring a minimum read copy number per mutation, given that some mutations may be filtered out through the use of such a threshold (Table S13), as recently suggested (Schmitt et al., 2024).”

Lines 153–157:
This is an intriguing result. However, the interpretation that a negative correlation between different de novo mutations supports the existence of two underlying cell lineages in each flower is not fully convincing. Please clarify the logic behind this conclusion. Specifically, explain how such a correlation pattern would arise from two distinct lineages and whether alternative explanations were considered.

The interpretation here hinges on the relationship between VAF (%) and cell variant frequency, CVF(%). In the previous version of the manuscript, we did not show sufficiently how CVF (%) is calculated. In the revised manuscript we have a new supplemental figure (Fig. S9) which shows how CVF (%) is calculated. The figure also shows that adding the estimates from pairs of our Group 1 and Group 2 markers all sum to near 100%. We provide the full raw data for the marker pair estimates in a new supplemental table (Table S8). The assumptions are presented in more detail now in an expanded part of the Methods section (Lines 512-530. We thank the reviewer for identifying this area for improvement.

Lines 159–170:
The authors present an interesting argument; however, it is difficult to draw definitive conclusions from data at the cell population level alone. Stem cells may be lost due to drift, which complicates lineage inference. To support their interpretation, the authors should consider the underlying stem cell population dynamics and discuss what types of dynamics could account for the observed mutation patterns. Incorporating a mathematical model could help bridge the gap between individual cellular behavior and population-level observations (e.g., Satake and Tomimoto, Trends in Genetics, 2024: https://www.sciencedirect.com/science/article/pii/S0168952524001070).

We thank the reviewer for the helpful comments regarding how to describe the dynamics. Briefly, we now have modified our manuscript to incorporate the perspectives of three recent articles (Tomimoto & Satake 2023, Satake et al. 2023, and Tomimoto & Satake 2024) that address models of expected cell lineage behavior (discussed above also). We found those articles to be incredibly thoughtful, circumspect and well-grounded. We particularly admire the efforts by those authors to establish a common testable framework that can explain not only the very low mutation rates observed in oak, but also the very high mutation rates observed in more recent papers with Poplar and in the recent tropical tree studies.

In our earlier paper, we included mutation rates for this particular focal tree (Wang et al. 2019). We are eager to report mutation rates again in the near future for this tree including a much larger set of samples in an upcoming paper, hopefully involving collaborative work with our colleagues who have models to test.

In addition, the resolution of Fig. 3A is low and should be improved for clarity.

Fixed. In Fig. 3A, we removed the small numbers as they were redundant with the color coding. We now say in the figure legend that the full data table is included in the supplement (Table S7).

Line 171:
Regarding cell abundance in L1 and L2 layers, please provide actual microscopic images, rather than illustrations, used in the estimation. Authors used published images, but environmental conditions are different, probably accession too. I suggest to collect images from the same individual used in this study. The method used to estimate cell abundances also needs to be clearly described.

We now include the microscope images as two new supplemental figures (Fig. S10 and S11). We have also retitled our methods section (Lines 533-553) describing these measurements as “Histological cross-sections measured for L1 and L2 cell proportions”

Specifically, we say in Lines 541-544 that “All images were longitudinal sections, apart from the leaf blades which were transverse sections. Tracings were made of each image, and cells were then counted in ImageJ 1.54 with settings: 8-bit and circularity > 0.3, with counts performed separately for the interior cells and the single outer layer of epidermal cells. Because Dermen and colleagues concluded that L2 and L3 are derived from a single underlying cell lineage, we grouped them together as the interior cell count and we focused on their combined percentage relative to L1 cells.”

Line 352:
Since GATK’s UnifiedGenotyper is known to yield a relatively high false positive rate, the reliability of SNV calls should be validated using additional tools such as GATK HaplotypeCaller, which is more commonly used in recent studies. In Fig. S3, it appears that both UnifiedGenotyper and HaplotypeCaller were used. Please clarify this point.

We thank the reviewer for this important point and we have extensively clarified the SNP identification methodology both in the Methods Section and in the supplemental figure (Fig. S3).

Specifically, we have added the following two new paragraphs (Lines 453-478) to the Methods:

“To extract putative single nucleotide polymorphic sites, we used the current standard Haplotype Caller in GATK 4.0 in combination with earlier use of UnifiedGenotyper (UG) in GATK3.5 (Fig. S3D), and only sites with a patch assembly mass greater than 20 (error rate 1%) were included. This resulted in a preliminary set of 1,888,276 SNV for filtering. The filtering pipeline for these SNV (Fig. S3E), consisted of the follow three main steps to remove: 1) SNVs representing parental heterozygosity, 2) local SNV regions with poor quality (poor read quality scores, three variants at SNV, strand bias, or SNV next to an INDEL) and 3) SNPs present in the controls or otherwise did not pass visual inspection. To remove parental variants, we remove sites with more than 2 alt reads found in any control (--max-cmp-depth 2) and removed sites with alt reads observed in more than 3 controls (--max-cmp-total 3). This filter removed 1,848,688 SNVs, leaving 39,588 SNV that were not due to parental differences. To remove sites with poor local quality, we filtered out sites with no sample with at least 5 reads (--min-supp-depth 5), missing in more than three controls (--max-cmp-miss 3), strand bias, or exhibiting strand bias. To remove SNPs that were adjacent to INDELs, we matched the SNVs and INDEL positions and removed SNVs with overlap. This filter removed 39,409 SNV, leaving 179 SNV with good general local site quality. Of these 179 SNPs, 109 occurred in SNV site clusters or exhibited poor overall read alignment and were excluded, leaving 70 high quality candidate de novo mutations in the flowers.

We then reprocessed the full set of variant candidate files using the currently standard approach of HaplotypeCaller in GATK (4.2.4.0) using the GVCF mode and merged the individual gvcfs into a vcf file containing only variant sites. Here, we filtered out unreliable SNVs using GATK VariantFiltration with the following filters: QD (Qual By Depth)<2.0, QUAL (Base Quality)<30.0, SOR (Strand Odds Ratio)>4.0, FS (Fisher Strand)>60.0, MQ (RMS Mapping Quality)<40.0, MQRankSum (Mapping Quality Rank Sum Test)<–12.5, ReadPosRankSum (Read Pos Rank Sum Test)<–8.0, following the standard in the literature (Satake et al. 2023).”

Line 354:
More specific filtering criteria for visual inspection of SNVs should be described. Fig. S3, which appears relevant, should be cited here.

Done. In addition to the new text mentioned above, we have revised Supplemental Figure S3 to show the filtering criteria more clearly. We clarify that our filters first removed parental heterozygosities, followed by abnormal local total read counts (too low, too high, biased strands, triallelic, or adjacent to indels), and finally visual inspection to identify unusual clustering (more than 3 per 150bp) or poor overall read alignment. We greatly appreciate the suggested revision.

Figure 2B, C: Please explain what "a" and "b" represent—presumably replicates? If so, please state this explicitly.

Fixed. Thank you. We modified the Results section (Lines 167-178), the Methods section (Lines 487-490), and the figure legend for Figure 2 to improve our wording relating to the twenty splitsamples to clarify that “a” and “b” to refer to the two independently genotyped subsamples.

Figure 2D, E:
Y-axis labels are missing in the bar plots. Please add them to improve readability.
Fixed. Thank you. We have added the Y-axis labels to the bar plots as suggested. We appreciate these changes which have substantially improved Fig. 2.

Data availability
It seems like accession number is still not obtained.
Our Read Archive repository accession number is PRJNA1277537. The reviewer link to access the dataset is as follows:

<https://dataview.ncbi.nlm.nih.gov/object/PRJNA1277537?reviewer=3bsjhlhv68eqd5j16hfaaps87h>

I also think that authors need to provide code used for analyses.

We provide some additional information in the Methods section (Lines 453-478). We will make all of the underlying analysis code available on Github.

Reviewer #2: This study by Ji et al. is one of the first attempts to describe which cell lineages lead to the development of which plant sub-part, following the accumulation of somatic mutations in a woody plant (peach). This approach complements and deepens the knowledge we have from histological studies, which suggests a two cell bottleneck at the onset of flower development. Using next-generation sequencing, the study examines the accumulation of nested mutations, offers valuable insights into the early segregation of cell lineages in flowers and lays the groundwork for further genomic explorations in plant developmental biology.

The authors collected micro-punch samples from two flowers and performed whole-genome sequencing to an average depth of 64.3x. They identified a total of 70 mutations: 20 in Flower #1 and 13 plus 37 in Flower #2. By analyzing mutations shared among different samples, they were able to investigate the phenomenon of sectoring. To evaluate the reliability of their somatic mutation calls, the authors split the samples into two batches, generating independent variant calls of the same genome. Their high replication rate for somatic mutations (97%) confirms that these mutations can be effectively tracked in the different flower parts. They also verified that the percent variant allele frequency (VAF%) was consistent between independent mutation calls.

They find, by focusing on two mutations present flower-wide and whose frequency is negatively correlated with each other, evidence in support of two underlying cell lineages giving rise to a flower. This is further confirmed by comparing mutations found in earlier (branch) tissues. Specifically, they identify branch-derived mutations present in both flowers, some of which correlate positively and others negatively with the flower-specific mutations. Based on these observations, the authors assign the mutations to two separate groups corresponding to two distinct cell lineages.To test whether each flower’s component cell lineages emerged from a single founder cell, the authors compared the VAF% in major tree branches to those in the flowers. In three out of four cases, the relative abundances of the groups’ mutations remained the same in the main tree and the flower, suggesting that frequencies did not change over the transition from branch to flower. When they plotted the frequency of these mutations over time (as the tree grew), they found that the proportion of group 2 cells tended to decrease. Finally, in Figure 5, they recapitulate the cell lineages contributions to flower development by following the emergence of mutations in the different sectors that they describe in Figure 1.

Overall summary :
I find this paper clever and relevant - an important contribution to the field of plant development and molecular biology. Their approach is clearly illustrated with figures and schemas, although some of them could be clearer and simpler (see comments below).

We thank the Reviewer for the helpful summary of the work and for also identifying areas for improvement.

Minor comments
L5: Remove redundant "whether" in "ask whether 1) whether radial sectors accumulate nested mutations."

Corrected in Line 5. Thank you.

L87-89 : the sampling and subsampling design has not been described and the term Flower #2 cannot be understood at that stage. Consider re-phrasing/giving more detail at this stage of the paper.

Done. Thank you. We have reworded that sentence (now Lines 108-109) to say “two flowers, hereafter Flower #1 and #2”

L104 : authors find a total of 70 new mutations between two flowers - this is a very high number of mutations. How does it compare to the number of mutations we would expect in annual tissues? I believe this is missing in the discussion.

We now include a new supplemental table (Table S13) which is a nearly complete list of all studies reporting somatic mutation identification in woody angiosperms, including sample sizes and tissues studied.

In the Discussion (Lines 350-361) we now say

“Thus, independently, our peach and their apricot datasets both suggest exceedingly low rates of point mutation in woody tissues of Prunus, on the order of one event per cell lineage per branch initiation. This estimate is in line with the findings of earlier studies in other woody trees (Schmid-Siegert et al., 2017; Plomion et al., 2018; Perez‐Roman et al., 2022; Duan et al., 2022; Orr et al.). However, these studies were all generally conducted on leaves. With respect to other studies, our mutation numbers per biological sample reported here for flowers (~0.7) are at the low end of the range observed in other studies of woody angiosperm tissues, where the average is around 4 mutations per sample, with substantial variation between studies (Table S13). Notably, none of the prior studies have explored differences among floral parts. Our relatively-stringent filtering of candidates in the mutation identification pipeline may also contribute to the low estimate. Future studies should not filter candidates by requiring a minimum read copy number per mutation, given that some mutations may be filtered out through the use of such a threshold (Table S13), as recently suggested (Schmitt et al., 2024).

Fig 1. C&D : the labels are too small to be read/informative.

Done. We have revised Fig. 1 to improve the font and resolution. Thank you.

Figure S3 filter criteria - very good chart. Are the filter criteria all exclusive ? i.e. if they meet the filter, they get filtered out ? I think that could be a bit more clear in the legend.

We now clarify Figure S3 to say that the criteria were applied sequentially.

Mutation #M42 is used as a focal/example mutation for Figure 2B, but what is the justification behind this choice ?

We now say in the Results (Lines 177-178) that “#M42 is presented because it was the most widespread de novo floral mutation in Flower #2 and present in all 20 split samples”

In the Fig. 2 legend we now say “focal mutation #M42, the only *de novo* mutation present in all subsamples”. We appreciate the suggestion.

Fig. 3. : The visual could be improved : the table and labels are blurry and the information could be conveyed in a clearer way with a bar plot for example. Overall, the quality of graphics is not consistent, make sure to export vector format figures to ensure high resolution.

We thank the reviewer for the suggestions. We have remade Fig. 3 with new graphics with higher resolution. We have added flower part information to two of the scatterplots. We believe these changes have improved the presentation.

Fig. 4. and L205 : can you explain a bit more how and why you choose the thresholds for Group 1 versus Group 2 mutations ?

We have revised Fig. 4F to show the threshold more clearly and box the eight mutations assigned to each group. The revised figure legend for Fig. 4F now reads as follows:

“Eight sectoring mutations assigned to Group 2 (#M53, #M57, #M38, #M6, #M7, #M35, #M41, and #M17) and eight assigned to Group 1 (#M4, #M2, #M46, #M56, #M9, #M40, #M25, #M25, #M52), respectively. Assignment based on maximum observed VAF (%) values (triangles) in carpel, sepals, or stamens, but not petals. Mutations ordered from highest to lowest max VAF(%). Threshold determined as max VAF (%) > 21.0% for Group 2 inclusion and VAF(%) < 21.0% for Group 1 inclusion. Threshold chosen because 21.0% was the maximum value observed for the two de novo flower-wide Group 1 mutations (#M11, #21) in carpels, sepals, or stamens. Box and whisker plots for #M11 and #M21 shown. Mutation values from petal samples were excluded due to the overlapping distributions (shown in Panel D). One outlier for #M11 in a stamen excluded. Raw data included (Table S8).”

We thank the Reviewer for suggesting this significant clarification to the figure.

Reviewer #3: Ji and colleagues use high-coverage short read sequencing to explore patterns of somatic mutations in two peach flowers. The importance of characterizing somatic mutations in floral tissue can be related to 1) understanding the number of founder cells that give rise to the developed flower and 2) determining to what extent somatic mutations are transmitted to progeny.

The authors present a very nice data set to explore these questions using both in-depth sampling of floral tissue and high depth of sequencing to uncover somatic mutations that may occur at low variant allele frequencies. The main findings of this work are that 1) somatic mutations are nested and can be traced to determine cell lineages, 2) that there are likely only two floral founder cells (one gives rise to L1 and one to L2), and 3) carpels do not share these mutations with the rest of the floral organs. Overall, the dataset and results presented are exciting and of interest of researchers in the field. I noted a few concerns below.

We thank Reviewer #3 for the feedback and the important suggestions.

1) The results presented in the manuscript depend heavily on the SNP calling methodology. I was disappointed that methods used for SNP calling only referenced “standard guidelines for screening somatic point mutations” (Barnell, 2019). This referenced article outlines a workflow for calling somatic mutations between tumor/normal sample pairs. If such a paired strategy was used then it must be more carefully detailed in the methods (i.e. what was the “normal” sample?). Similarly, Figure S3 outlines criteria 4 for filtering and some additional clarifications are needed.

We thank the Reviewer for suggesting these improvements. We have thoroughly revised Fig. S3 and added a significant amount of detail to the Methods section in three paragraphs (Lines 442-478).

Specifically, we now say the following:

“Sequencing and data processing steps followed standard approaches for point mutation identification (Fig. S3). Amplification products were sequenced by Illumina Xten platform (BGI-Shenzhen) and mapped to the v2.0 peach reference genome (Verde et al., 2013). The proportion of high-quality bases (>20) exceeded 92% with an average clean read depth of 64.3x (Table S1). We created the assemblies with BWA-mem 0.7.10-r789, sorted and removed PCR repeats with MarkDuplicates in Picard (v1.114), and locally reassembled with IndelRealigner in RealignerTargetCreator (Fig. S3C). To identify de novo mutations that arose specifically in each flower, we used two main types of controls. These controls included 1) samples from the other flower and 2) a published whole-canopy collection of leaf samples from the four main branches of the tree (Wang et al. 2019). A de novo floral mutation is thus defined as being present in that flower but absent from all other samples from the tree.

To extract putative single nucleotide polymorphic sites, we used the current standard Haplotype Caller in GATK 4.0 in combination with earlier use of UnifiedGenotyper (UG) in GATK3.5 (Fig. S3D), and only sites with a patch assembly mass greater than 20 (error rate 1%) were included. This resulted in a preliminary set of 1,888,276 SNV for filtering. The filtering pipeline for these SNV (Fig. S3E), consisted of the follow three main steps to remove: 1) SNVs representing parental heterozygosity, 2) local SNV regions with poor quality (poor read quality scores, three variants at SNV, strand bias, or SNV next to an INDEL) and 3) SNPs present in the controls or otherwise did not pass visual inspection. To remove parental variants, we remove sites with more than 2 alt reads found in any control (--max-cmp-depth 2) and removed sites with alt reads observed in more than 3 controls (--max-cmp-total 3). This filter removed 1,848,688 SNVs, leaving 39,588 SNV that were not due to parental differences. To remove sites with poor local quality, we filtered out sites with no sample with at least 5 reads (--min-supp-depth 5), missing in more than three controls (--max-cmp-miss 3), strand bias, or exhibiting strand bias. To remove SNPs that were adjacent to INDELs, we matched the SNVs and INDEL positions and removed SNVs with overlap. This filter removed 39,409 SNV, leaving 179 SNV with good general local site quality. Of these 179 SNPs, 109 occurred in SNV site clusters or exhibited poor overall read alignment and were excluded, leaving 70 high quality candidate de novo mutations in the flowers.

We then reprocessed the full set of variant candidate files using the currently standard approach of HaplotypeCaller in GATK (4.2.4.0) using the GVCF mode and merged the individual gvcfs into a vcf file containing only variant sites. Here, we filtered out unreliable SNVs using GATK VariantFiltration with the following filters: QD (Qual By Depth)<2.0, QUAL (Base Quality)<30.0, SOR (Strand Odds Ratio)>4.0, FS (Fisher Strand)>60.0, MQ (RMS Mapping Quality)<40.0, MQRankSum (Mapping Quality Rank Sum Test)<–12.5, ReadPosRankSum (Read Pos Rank Sum Test)<–8.0, following the standard in the literature (Satake et al. 2023). ”

Criteria 4) SNV is parental heterozygosity (in all samples). I understood the genome was diploid. If mapping to a diploid genome then parental heterozygozity should be reflected as differences between the haplotypes (not variants identified via mapping of short reads to the reference). Is the reference genome assembled from the same tree? Please provide clarification.

We mapped to the v2.0 peach reference genome (Verde et al., 2013) we find that it is 99.99% identical in sequence to one of the parents of the tree. The identity of the other parent is unknown, but has roughly 1.8 million SNP differences, which were identified and excluded.

Criteria 7/8) 3 or more SNV reads across all controls. 2 or more SNV reads in any single control. What were the control samples? Please clarify. Most importantly, Why were 680 of 750 (80%) SNVs remaining after filtering based on criteria 1-11 excluded by visual check? What were the criteria for exclusion? These 680 variants were characterized as true negatives in S6A. How was this determined? While the results from the small number of high confidence variants (70) were intriguing. I am very concerned about the exclusion of 80% of variants at this stage.

We thank the Reviewer. We now say more clearly in Lines 447-451 that “ To identify de novo mutations that arose specifically in each flower, we used two main types of controls. These controls included 1) samples from the other flower and 2) a published whole-canopy collection of leaf samples from the four main branches of the tree (Wang et al. 2019). A de novo floral mutation is thus defined as being present in that flower but absent from all other samples from the tree.”

2) One of the key findings of the manuscript is that there are two cell lineages marked by somatic variants in each flower. Convincing evidence was presented to suggest that there is likely just two progenitor cells, one each for the L1 and L2 layers and that the variant frequency for mutations in each lineage are anticorrelated. The lack of shared somatic mutations in the carpels was concerning. The authors provide some argument that carpels are distinct, with their own vasculature, and so this could be an explanation for their observations. Please provide additional supporting evidence or references to support the hypothesis that the carpel cell lineage is not expected to derive from one of the two floral founder cells.

We apologize for the misunderstanding. We DO think that the carpel derives from these floral founder cells. To clarify this, we have modified the Abstract (Lines 11-12) to say “Both carpels possessed two flower-wide de novo mutations, confirming that their genetic origin does not precede the floral anlagen.” We also show this in Fig. 5 where the cell lineages accumulate the mutations before forming the carpel tissue.

3) The authors also observe that carpel cell lineages do not share mutations with the rest of the floral organs and hypothesize that female gametes are protected from mutation. What about the male gametes? Is there evidence to support reduced mutation in female vs male gametes in peach? Or other tree crops?

We do not know of any published data on this topic, but it is an interesting question. Because we focused on two carpels, our sample size was not sufficient for us to address the question here.

4) Only L2 associated mutations were identified in surveyed progeny. This is evidence that some somatic mutations can be transmitted. I was surprised that these mutations were not fixed in the progeny and occurred at a range of variant allele frequencies. Variants transmitted through the egg cell or sperm cell should be fixed (or at a VAF of 0.5 for haploid reference genomes).

We have revised the VAF diagram shown for the progeny (Fig. S12F) to emphasize the pattern, which is derived from Xten sequence data. We provide the raw read data in Table S11 and now state that raw data are provided in the figure legend for Fig. S12. In some cases, a deviation in read counts from 50:50 was observed and is not unexpected due to sampling variation as we report with our split subsamples (Table S6).” We think these changes have improved the manuscript and we thank the reviewer for the valuable suggestions that have improved the revised manuscript.

Not clear if the underlying data has been uploaded to NCBI SRA

Our NCBI SRA is PRJNA1277537 can be accessed with the following link for reviewers: <https://dataview.ncbi.nlm.nih.gov/object/PRJNA1277537?reviewer=3bsjhlhv68eqd5j16hfaaps87h>. We thank the reviewer for the helpful suggestions.
